# Women’s marital status and use of family planning services across male- and female-headed households in low- and middle-income countries

**DOI:** 10.7189/jogh.13.04015

**Published:** 2023-03-03

**Authors:** Franciele Hellwig, Ghada E Saad, Andrea Wendt, Aluísio JD Barros

**Affiliations:** 1 International Center for Equity in Health, Federal University of Pelotas, Pelotas, Rio Grande do Sul, Brazil; 2 Postgraduate Program in Epidemiology, Federal University of Pelotas, Pelotas, Rio Grande do Sul, Brazil; 3 Faculty of Health Sciences, Department of Epidemiology and Population Health, American University of Beirut, Beirut, Lebanon

## Abstract

**Background:**

As more households are being led by women, who are often seen as disadvantaged, more attention is being given to the potential association of female household headship with health. We aimed to assess how demand for family planning satisfied by modern methods (mDFPS) is associated with residence in female or male headed households and how this intersects with marital status and sexual activity.

**Methods:**

We used data from national health surveys carried out in 59 low- and middle-income countries between 2010 and 2020. We included all women aged 15 to 49 years in our analysis, regardless of their relationship with the household head. We explored mDFPS according to household headship and its intersectionality with the women’s marital status. We identified households as male-headed households (MHH) or female-headed households (FHH), and classified marital status as not married/in a union, married with the partner living in the household, and married with the partner living elsewhere. Other descriptive variables were time since the last sexual intercourse and reason for not using contraceptives.

**Results:**

We found statistically significant differences in mDFPS by household headship among reproductive age women in 32 of the 59 countries, with higher mDFPS among women living in MHH in 27 of these 32 countries. We also found large gaps in Bangladesh (FHH = 38%, MHH = 75%), Afghanistan (FHH = 14%, MHH = 40%) and Egypt (FHH = 56%, MHH = 80%). mDFPS was lower among married women with the partner living elsewhere, a common situation in FHH. The proportions of women with no sexual activity in the last six months and who did not use contraception due to infrequent sex were higher in FHH.

**Conclusions:**

Our findings indicate that a relationship exists between household headship, marital status, sexual activity, and mDFPS. The lower mDFPS we observed among women from FHH seems to be primarily associated with their lower risk of pregnancy; although women from FHH are married, their partners frequently do not live with them, and they are less sexually active than women in MHH.

Several global initiatives prioritize achieving universal access to family planning services by 2030 [1-4]. Much progress has already been made in increasing demand for family planning satisfied by modern methods (mDFPS) in low- and middle-income countries over the past decades [5,6]. However, inequalities between and within countries still exist, especially regarding socioeconomic factors such as wealth, area of residence, and women’s education, with lower levels of mDFPS being documented for adolescents, women living in rural areas, the poorer and among women with lower levels of education [7-9].

mDFPS is also one of the key indicators of the Sustainable Development Goals, related to both health and gender equality. Although access to family planning services has been documented as an important factor in increasing women's participation in labor and economic empowerment [10], evidence of the effects of family planning on women’s empowerment is inconsistent [11]. One of the most studied domains of women’s empowerment is the decision-making power of women over household decisions, which has been associated with increased use of contraceptives [12]. Women’s decision-making may be influenced by inter-household dynamics as well as who heads the household [13]. Male-headed households (MHHs) may be more restrictive of women’s autonomy and decision-making power, while women in female-headed households (FHHs) have more control over their decisions. Nevertheless, depending on the circumstances that led to a household being headed by a female, women in these households may be subjected to increased socioeconomic and psychological vulnerability due to limited employment opportunities and inequitable income, especially if no other males are present in the household, consequently limiting their resources [14,15].

As the proportion of FHHs increased in recent years [14,16], more studies have explored the potential association of female household headship and contraceptive use. However, these studies are often restricted to a country or a specific age-range [17,18]. There is evidence of higher unmet need for contraception among young women aged 15-24 years old in female-headed households in African countries [17]. A study in Ghana found no difference in mDFPS among reproductive age women living in MHHs and FHHs [18]. Besides women’s age, other characteristics are related to household headship and mDFPS, such as marital status and polygyny, both of which are associated with female headship and frequency of sexual intercourse [19-23].

Given the persistent inequalities in family planning documented between and within countries and the insufficient evidence on the association between mDFPS and household headship, we aimed to explore the differences in demand for family planning satisfied by modern contraceptives among reproductive age women residing in MHHs and FHHs and how this intersects with women’s marital status and sexual activity in low- and middle-income countries.

## METHODS

We used publicly available data from the most recent Demographic and Health Surveys (DHSs) [24,25] carried out from 2010 to 2020. We included all surveys that collected data on family planning for women aged 15-49 years. We used data from 59 low- or middle-income countries. We based the analyses on all sexually active women in their reproductive age (15-49 years old), residing in the sampled households regardless of their relationship with the household head, meaning we included women that were the heads, spouses, daughters, or women not related to the household head in the analyses. We did not set restrictions on marital status, except in Afghanistan, Bangladesh, Pakistan, Turkey, and all surveys from the Middle East and North African region – Egypt, Jordan, and Yemen – from where we included only women who were ever married or in a union because the information on contraception was not collected for never-married women.

### Outcome

Our main outcome is mDFPS, defined as the proportion of women in need of contraception who were using (or whose partner was using) a modern contraceptive method. We considered a woman as in need of contraception if she was sexually active, fecund, and did not want to become pregnant within the next two years, or if she was unsure about whether or when she wanted to become pregnant. We also considered pregnant women with a mistimed or unintended pregnancy as in need of contraception. We considered women as sexually active if they were married or living with a partner, or if they were not married but reported having had sexual intercourse in the month preceding the interview. We classified methods as modern if they were medical procedures or technological products [26], including oral contraceptive pills, injections, male and female condoms, diaphragms, spermicidal agents, emergency contraception, intrauterine devices (IUD), implants, and sterilization (female or male).

### Statistical analyses

We conducted descriptive analysis at the country and regional levels. We used the UNICEF classification for world regions (Latin America and the Caribbean, South Asia, East Asia and the Pacific, Europe and Central Asia, Middle East and North Africa, Eastern and Southern Africa, and West and Central Africa) [27]. Using our selected variables related to the household and all women of reproductive ages, we carried out descriptive analyses to illustrate the characteristics of the household heads, the households, and all women aged 15-49 within these households, by country and region.

We then calculated mDFPS among all women of reproductive age by the household head’s sex for each country and region. We calculated the differences in the percentage of women reporting mDFPS by sex of the household head and presented them as equiplots at the country level. We then analyzed the mDFPS for women of reproductive age by sex of the household head and by our categories of marital status by regional levels. Country-level estimates of mDFPS, sample sizes, and 95% confidence intervals by sex of the household head and marital status are presented in Table S1 in the **Online Supplementary Document**.

Using the variable concerned with the reason for non-use of modern contraceptives, we generated the percentages of women who selected each of the reasons for non-use, stratified regionally by household headship and marital status.

We performed all analyses using Stata software version 16.1 (StataCorp LLC, College Station, TX) and adjusted for the sample design, including sample weights, clusters, and strata. All analyses relied on publicly available anonymized databases. Institutions and national agencies in each country gave ethical approval for the surveys.

## RESULTS

We included 738 836 women between the ages of 15 and 49 years from 59 countries. The countries included in our analysis represent 17% of the Middle East and North African (MENA) countries, 71% of the countries in Eastern and Southern Africa, 75% of those West and Central Africa, 23% of those Europe and Central Asia, 75% of those South Asia, 43% of those East Asia and the Pacific, and 18% of the countries in Latin America and the Caribbean.

### Demographic characteristics and sexual activity of women residing in male and female headed household

The average median age of the household head was higher in FHH, especially in MENA countries, where the average median age of the household head was 46 years in MHH and 57 years in FHH. All regions presented large differences in the proportion of currently married household heads according to the sex of the head, at least 50 percentage points higher in MHH than in FHH. The highest difference was observed in West & Central Africa, where 96% of the male heads were currently married compared to only 12% of the female heads. The proportion of households with other women who were at least 15 years of age was consistently higher in FHH (**Table 1**). For example, in West and Central Africa, the proportion of other women 15 years or older is 63% in FHH and 44% in MHH; in Latin America and the Caribbean, this proportion ranges from 65% in FHH and 47% in MHH. The proportions between FHH and MHH in South Asia are similar, at 76% and 74%, respectively (**Table 1**).

**Table 1:** Descriptive characteristics of households and women residing in these households, aged 15-49 years old, by sex of household head and region in 59 low- and middle-income countries*

		Household head			All sampled women of reproductive age					
**Region**	**Household headship**	**Median age (in years)**	**Currently married (%)**	**Other female 15+ years (%)†**	**Median age (in years)**	**Currently married (%)**	**Husband/partner present (%)**	**Polygyny (%)**	**Sexual intercourse in the last month (%)**	**No sexual intercourse in the last six months (%)**
West and Central Africa	MHH	45	88.1	44.2	29	74.6	92.1	30.6	66.6	9.3
	FHH	48	37.9	63.3	29	42.1	30.0	33.6	36.1	26.6
Eastern and Southern Africa	MHH	43	86.0	35.5	28	70.0	90.0	10.4	73.4	7.2
	FHH	47	31.8	51.4	29	39.9	40.2	22.0	34.7	27.8
Middle East and North Africa	MHH	46	96.0	61.9	31	85.9	93.4	4.5	89.1	4.1
	FHH	57	11.8	72.3	33	39.9	44.9	6.1	26.5	60.0
Europe and Central Asia	MHH	53	92.5	56.4	31	72.1	95.5	1.9	80.4	6.4
	FHH	59	19.5	61.8	31	46.1	76.2	6.2	50.7	28.2
South Asia	MHH	48	93.6	74.4	30	86.0	90.8	2.0	76.3	6.0
	FHH	51	35.6	75.7	30	71.1	46.1	2.1	40.7	28.2
East Asia and the Pacific	MHH	47	91.5	55.6	31	70.5	94.6	35.0	72.9	8.6
	FHH	54	22.8	72.4	30	69.8	65.4	30.0	39.7	38.1
Latin America and the Caribbean‡	MHH	47	86.5	47.0	30	40.6	95.4	8.2	75.3	7.3
	FHH	49	34.3	65.4	30	32.6	57.7	20.6	42.9	27.0

MHH – male-headed households, FHH – female-headed households

*Source: Demographic and Health Survey 2010-2019.

†Other female 15+ years includes usual female residents aged 15 y or more who were not the head or the wife of the head.

‡In Latin America and the Caribbean, information on polygyny is available only for Haiti.

The median age of all the women aged 15-49 years in our sample was 30 years in both MHH and FHH. Across all regions, most of the women of reproductive age living in MHH were married or in union; among them, more than 90% were living in the same household as their partners/husbands. However, a large proportion of women of reproductive age living in FHH were not married nor living with a partner; among those who were married, more than half were not living with their partners/husbands. Higher proportions of currently married women living with their partners/husbands in FHH were observed in Europe and Central Asia (76%), East Asia and the Pacific (65%), and Latin America and the Caribbean (58%). We found the highest proportion of married women whose husbands lived elsewhere in West and Central Africa, where 42% of the women of reproductive age were married and only 30% of them lived in the same household as their partners (**Table 1**).

West and Central Africa also had one of the highest prevalence of polygyny, 34% in FHH and 31% in MHH. Polygyny was also high in East Asia and the Pacific, where 30% of married women were in a polygynous relationship in FHH and 35% in MHH. This was the only region where the proportion of women whose husband had other wives was higher in MHH than in FHH. The lowest prevalence of polygyny was observed in South Asia, 2% in both MHH and FHH and in Europe and Central Asia, within MHH (2%) (**Table 1**).

**Table 1** also presents data regarding all women’s sexual activity. There was a large difference between women in MHH and those in FHH, where reports of sexual intercourse in the last month were higher in MHH compared to FHH across all regions. A small proportion of the women in MHH declared that their last sexual intercourse was more than six months ago, while this proportion was much higher among women in FHH (**Table 1**).

Country-level estimates of the descriptive characteristics of households and women of reproductive age are presented in Table S2 in the **Online Supplementary Document**.

### Disparities in mDFPS

**Figure 1:**
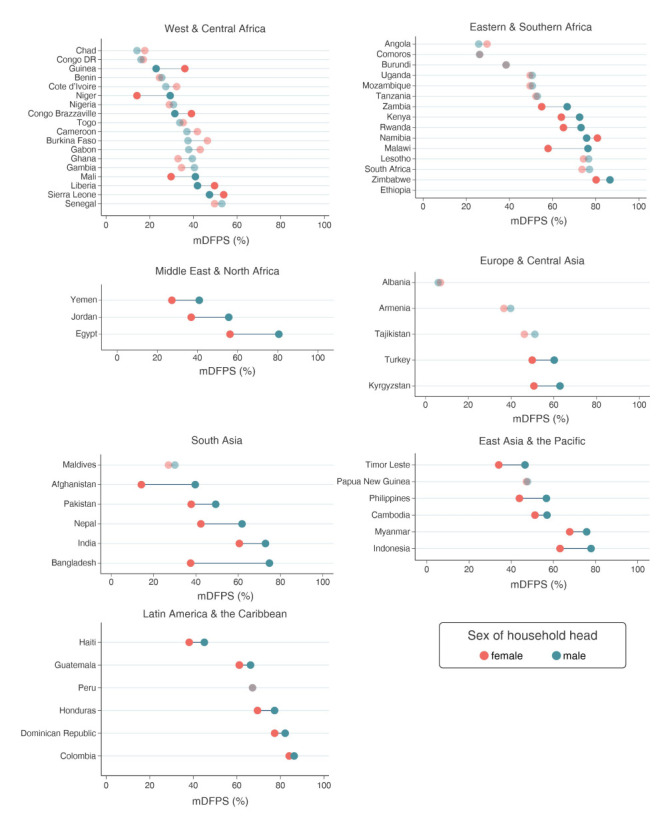
Percentage of women, aged 15-49 years, living in female and male headed households who had their mDFPS by household headship and region in 59 low- and middle-income countries. Source: DHS, 2010-2019. We ordered the countries according to the mDFPS in male headed households. In Afghanistan, Bangladesh, Egypt, Jordan, Pakistan, Turkey, and Yemen the sample is restricted to ever married women. Darker dots indicate that the differences are statistically significant.

The level of mDFPS among our sample of women of reproductive age in each country by sex of the household head is presented in **Figure 1**. Our results indicate important differences in mDFPS by sex of the household head, with higher levels in MHH in most countries. Larger gaps were found in Bangladesh, where mDFPS was 37.5% in FHH and 74.8% in MHH, Afghanistan (14.2% in FHH and 39.7% in MHH), and Egypt (56.2% in FHH and 80.6% in MHH) (**Figure 1**).

In most African countries, there was no difference between women residing in FHH compared to women in MHH. The exception was Guinea, where the difference was 13.2 percentage points (36.1% in FHH and 22.9% in MHH) (**Figure 1**).

**Figure 2:**
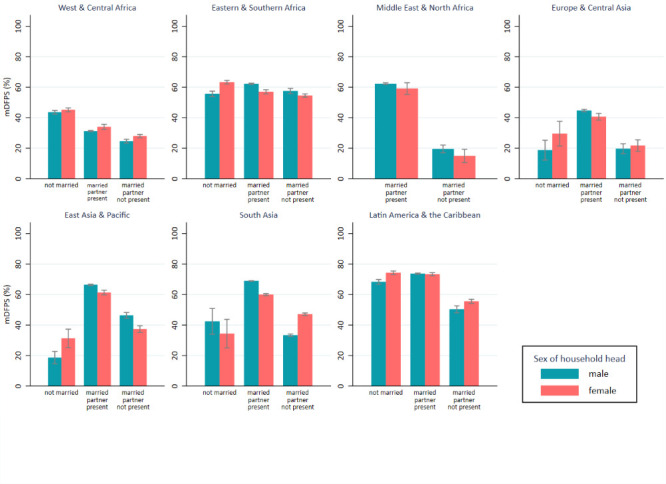
mDFPS among women aged 15-49 years by sex of the household head, marital status, and presence of the husband/partner in the household in 59 low- and middle-income countries. Source: DHS, 2010-2019.

When considering marital status and presence of the partner/husband in the household, we observed lower levels of mDFPS in both MHH and FHH among women who were married but the partner/husband resided elsewhere compared to women whose partners/husbands were present in the household. Among nonmarried women, mDFPS was similar to that of women married and living with the partner/husband in Latin America and the Caribbean and in Eastern and Southern Africa, while it was higher in West and Central Africa and lower in all Asian regions (**Figure 2** and **Table 2**).

**Table 2:** mDFPS among women aged 15-49 years old in need of family planning, stratified by marital status, presence of the partner/husband in the household and sex of household head in 59 low- and middle-income countries*

		mDFPS			
		**FHH**		**MHH**	
**Country**	**Marital status**	**% (95% CI)**	**N**	**% (95% CI)**	**N**
**West and Central Africa**					
Benin (2017)	Not married	30.5 (24.5-37.2)	287	28.0 (23.6-32.8)	421
	Married – partner present	24.0 (18.9-29.9)	264	25.7 (24.0-27.4)	4270
	Married – partner not present	21.6 (18.2-25.6)	563	19.1 (14.4-25.0)	243
Burkina Faso (2010)	Not married	72.4 (61.3-81.3)	84	62.0 (53.1-70.1)	231
	Married – partner present	49.8 (33.3-66.3)	42	36.5 (34.6-38.4)	4918
	Married – partner not present	38.9 (32.2-46.0)	312	35.1 (27.6-43.4)	245
Cameroon (2018)	Not married	47.9 (43.4-52.5)	564	56.4 (50.5-62.2)	549
	Married – partner present	40.4 (32.6-48.7)	227	33.3 (30.8-36.0)	2683
	Married – partner not present	28.6 (22.6-35.5)	237	30.7 (23.2-39.4)	135
Chad (2014)	Not married	27.2 (19.0-37.4)	139	21.1 (14.0-30.5)	144
	Married – partner present	12.2 (6.8-21.0)	107	14.1 (12.2-16.1)	3160
	Married – partner not present	15.6 (11.0-21.5)	332	10.5 (6.5-16.4)	191
Congo Brazzaville (2011)	Not married	44.2 (38.4-50.2)	614	45.2 (39.5-51.1)	661
	Married – partner present	28.8 (19.9- 39.6)	143	28.9 (26.6-31.3)	3510
	Married – partner not present	31.6 (23.1-41.5)	222	31.8 (19.8-46.8)	93
Congo DR (2013)	Not married	21.1 (16.5-26.5)	423	24.5 (20.1-29.7)	693
	Married – partner present	16.2 (10.7-23.9)	228	15.1 (13.3-17.0)	4614
	Married – partner not present	14.8 (10.7-20.2)	677	9.8 (6.0-15.4)	299
Côte d'Ivoire (2011)	Not married	39.7 (33.3-46.6)	375	33.4 (29.1-38.0)	611
	Married – partner present	25.3 (16.8-36.3)	84	26.9 (24.3-29.7)	2331
	Married – partner not present	22.6 (16.0-30.9)	226	14.3 (9.7-20.6)	217
Gabon (2012)	Not married	50.7 (42.1-59.3)	696	58.2 (51.2-64.8)	529
	Married – partner present	33.4 (24.0-44.4)	179	32.6 (29.7-35.7)	1833
	Married – partner not present	34.3 (25.7-44.0)	403	30.6 (20.5-42.9)	163
Gambia (2019)	Not married	67.5 (48.6-82.0)	44	27.6 (12.9-49.5)	43
	Married – partner present	52.3 (43.6-60.8)	159	45.0 (41.9-48.1)	2199
	Married – partner not present	22.0 (16.3-29.2)	341	23.2 (18.9-28.1)	544
Ghana (2014)	Not married	32.9 (27.2-39.2)	451	37.7 (29.2-47.1)	181
	Married – partner present	39.0 (27.5-52.0)	97	40.3 (37.4-43.4)	2342
	Married – partner not present	31.7 (26.9-37.0)	474	22.7 (15.2-32.5)	97
Guinea (2018)	Not married	57.3 (48.4-65.8)	162	55.6 (48.4-62.7)	265
	Married – partner present	8.7 (3.9-18.0)	88	17.9 (15.2-20.8)	2034
	Married – partner not present	31.0 (22.4-41.1)	206	30.3 (23.0-38.8)	216
Liberia (2019)	Not married	56.3 (51.1-61.4)	732	48.2 (40.4-56.1)	448
	Married – partner present	37.9 (32.3-43.9)	380	40.6 (36.9-44.5)	1828
	Married – partner not present	47.4 (38.3-56.8)	179	33.2 (21.6-47.2)	74
Mali (2018)	Not married	35.4 (22.4-51.1)	61	41.5 (34.2-49.2)	190
	Married – partner present	34.8 (26.3-44.3)	181	41.4 (38.5-44.5)	2991
	Married – partner not present	23.8 (17.2-31.8)	209	28.4 (20.1-38.4)	139
Niger (2012)	Not married	54.5 (22.3-83.4)	14	0	4
	Married – partner present	23.0 (12.0-39.5)	35	31.1 (28.4-33.9)	2457
	Married – partner not present	10.7 (7.0-16.2)	254	9.9 (6.2-15.4)	210
Nigeria (2018)	Not married	33.7 (28.8-38.9)	695	29.7 (25.5-34.4)	616
	Married – partner present	25.6 (20.5-31.3)	300	31.2 (29.7-32.8)	8993
	Married – partner not present	25.6 (21.9-29.8)	673	24.6 (20.2-29.5)	371
Senegal (2019)	Not married	17.4 (4.4-49.0)	12	30.8 (10.7-62.4)	11
	Married – partner present	65.3 (55.7-73.7)	251	57.6 (54.1-61.1)	1581
	Married – partner not present	42.3 (36.5-48.4)	477	36.9 (32.9-41.1)	438
Sierra Leone (2019)	Not married	59.8 (55.5-63.9)	794	60.3 (56.1-64.4)	943
	Married – partner present	47.5 (41.4-53.7)	359	44.6 (42.2-47.0)	3213
	Married – partner not present	49.5 (44.9-54.0)	552	37.7 (32.0-43.8)	352
Togo (2013)	Not married	51.6 (44.7-58.5)	250	41.0 (34.5-47.8)	247
	Married – partner present	22.9 (14.2-34.9)	73	33.8 (31.5-36.2)	2693
	Married – partner not present	28.1 (23.6-33.2)	443	20.0 (14.1-27.6)	150
**Eastern and Southern Africa**					
Angola (2015)	Not married	37.3 (31.1-44.0)	635	37.3 (31.0-44.1)	541
	Married – partner present	27.1 (21.7-33.4)	523	24.2 (21.0-27.6)	3135
	Married – partner not present	17.3 (11.4-25.4)	293	18.0 (10.4-29.2)	159
Burundi (2016)	Not married	50.4 (37.5-63.1)	67	43.4 (29.0-58.9)	44
	Married – partner present	42.5 (33.2-52.4)	151	38.5 (36.5-40.5)	4883
	Married – partner not present	36.1 (32.3-40.1)	613	28.0 (16.6-43.3)	51
Comoros (2012)	Not married	33.4 (18.4-52.7)	43	35.2 (12.5-67.4)	36
	Married – partner present	24.2 (18.6-30.9)	360	25.1 (21.9-28.5)	1011
	Married – partner not present	28.3 (22.3-35.2)	206	33.5 (23.4-45.3)	109
Ethiopia (2016)	Not married	65.2 (51.2-76.9)	115	64.3 (40.7-82.6)	34
	Married – partner present	64.2 (55.8-71.9)	332	61.6 (58.5-64.7)	5125
	Married – partner not present	45.0 (37.4-52.8)	373	36.9 (23.1-53.4)	119
Kenya (2014)	Not married	64.9 (58.2-71.0)	421	71.2 (58.7-81.1)	114
	Married – partner present	58.5 (51.3-65.3)	258	72.9 (71.0-74.6)	4885
	Married – partner not present	64.9 (61.5-68.2)	1294	62.1 (51.2-71.8)	141
Lesotho (2014)	Not married	78.9 (71.3-84.9)	295	72.6 (61.8-81.3)	125
	Married – partner present	71.1 (61.3-79.2)	152	79.4 (76.4-82.1)	1450
	Married – partner not present	70.8 (63.9-76.9)	243	73.4 (70.0-76.5)	994
Malawi (2015)	Not married	53.7 (47.7-59.5)	455	47.1 (39.0-55.4)	260
	Married – partner present	74.1 (70.0-77.9)	591	78.1 (77.1-79.2)	10 250
	Married – partner not present	51.9 (48.1-55.7)	1276	53.9 (47.5-60.1)	450
Mozambique (2015)	Not married	58.2 (51.0-65.0)	294	67.1 (59.6-73.7)	172
	Married – partner present	45.0 (37.9-52.3)	267	49.2 (45.6-52.9)	1642
	Married – partner not present	45.1 (38.6-51.7)	291	44.9 (33.0-57.5)	91
Namibia (2013)	Not married	86.3 (83.4-88.8)	937	78.3 (73.1-82.7)	388
	Married – partner present	73.3 (68.1-78.0)	389	75.8 (73.1-78.2)	1474
	Married – partner not present	74.3 (68.7-79.2)	358	63.4 (51.0-74.3)	76
Rwanda (2019)	Not married	60.5 (53.0-67.5)	259	39.7 (30.5-49.8)	105
	Married – partner present	78.7 (70.8-84.9)	199	74.0 (72.5-75.4)	4954
	Married – partner not present	62.1 (57.4-66.5)	523	68.6 (57.1-78.3)	78
South Africa (2016)	Not married	72.8 (69.2-76.1)	1234	72.5 (65.9-78.2)	432
	Married – partner present	79.7 (72.1-85.6)	215	78.6 (75.7-81.1)	1599
	Married – partner not present	73.0 (66.0-79.0)	260	68.7 (54.3-80.2)	46
Tanzania (2015)	Not married	59.8 (53.6-65.8)	452	55.3 (48.2-62.2)	277
	Married – partner present	46.7 (38.8-54.8)	260	53.2 (50.9-55.5)	4341
	Married – partner not present	43.3 (34.5-52.5)	221	41.3 (32.0-51.3)	143
Uganda (2016)	Not married	58.8 (53.5-64.0)	495	52.9 (45.5-60.2)	261
	Married – partner present	48.3 (42.4-54.1)	416	50.7 (48.9-52.5)	5935
	Married – partner not present	45.3 (41.8-48.8)	965	44.4 (36.6-52.5)	239
Zambia (2018)	Not married	50.6 (44.9-56.3)	437	48.6 (41.7-55.6)	281
	Married – partner present	67.8 (60.3-74.5)	176	68.3 (66.3-70.2)	4669
	Married – partner not present	53.8 (47.1-60.4)	313	52.4 (43.6-61.1)	140
Zimbabwe (2015)	Not married	77.3 (71.2-82.4)	253	67.8 (54.2-78.9)	54
	Married – partner present	86.4 (82.4-89.6)	575	87.7 (86.1-89.1)	3031
	Married – partner not present	77.2 (73.9-80.2)	961	74.1 (64.4-81.9)	180
**Middle East and North Africa**					
Egypt (2014)	Not married	NA	NA	NA	NA
	Married – partner present	NA	NA	NA	NA
	Married – partner not present	NA	NA	NA	NA
Jordan (2017)	Not married	NA	NA	NA	NA
	Married – partner present	47.4 (33.3-61.9)	135	55.9 (54.1-57.7)	8558
	Married – partner not present	25.5 (15.0-39.8)	125	39.7 (30.4-49.8)	178
Yemen (2013)	Not married	NA	NA	NA	NA
	Married – partner present	52.4 (41.5-63.0)	111	43.1 (41.2-44.9)	8550
	Married – partner not present	11.6 (6.1-21.1)	179	18.6 (15.1-22.7)	845
**Europe and Central Asia**					
Albania (2017)	Not married	8.7 (3.5-19.8)	142	7.3 (3.8-13.4)	173
	Married – partner present	5.9 (3.9-8.9)	384	5.9 (4.9-7.1)	3813
	Married – partner not present	8.1 (4.8-13.4)	164	5.6 (3.0-10.2)	168
Armenia (2015)	Not married	89.7 (50.0-98.7)	6	100	4
	Married – partner present	38.3 (33.1-43.9)	475	40.4 (38.1-42.7)	2084
	Married – partner not present	25.8 (16.4-38.0)	102	14.0 (6.0-29.6)	53
Kyrgyzstan (2012)	Not married	37.9 (24.2-53.9)	72	62.7 (34.9-84.0)	21
	Married – partner present	60.7 (53.9-67.0)	306	64.9 (62.2-67.4)	2369
	Married – partner not present	26.1 (15.8-39.8)	87	12 (6.7-20.5)	92
Tajikistan (2017)	Not married	49.1 (13.3-85.8)	4	4.4 (0.5-31.0)	4
	Married – partner present	48.4 (42.9-53.8)	490	53.3 (50.7-55.8)	3103
	Married – partner not present	38.8 (28.5-50.2)	144	29.1 (22.8-36.2)	292
Turkey (2013)	Not married	NA		NA	
	Married – partner present	NA		NA	
	Married – partner not present	NA		NA	
**South Asia**				0	
Afghanistan (2015)	Not married	NA		NA	
	Married – partner present	19.9 (6.9-45.3)	88	40.1 (38.2-42.1)	13 033
	Married – partner not present	4.0 (1.2-12.7)	49	18.5 (10.8-29.8)	286
Bangladesh (2017)	Not married	NA		NA	
	Married – partner present	70.8 (65.1-76.0)	362	78.4 (77.4-79.3)	11378
	Married – partner not present	28.4 (25.4-31.6)	1330	31.0 (27.4-34.8)	948
India (2015)	Not married	80.6 (62.1-91.3)	71	55.7 (40.2-70.2)	85
	Married – partner present	65.0 (63.9-66.1)	18 866	74.3 (74.1-74.6)	298 046
	Married – partner not present	52.7 (51.5-54.0)	10 997	35.7 (34.5-37.0)	11 632
Maldives (2016)	Not married	15.2 (6.1-33.1)	67	2.6 (0.8-8.3)	48
	Married – partner present	30.5 (26.6-34.7)	866	32.4 (28.4-36.7)	1297
	Married – partner not present	19.8 (15.3-25.4)	304	20.7 (14.5-28.6)	181
Nepal (2016)	Not married	NA		77.0 (22.9-97.4)	3
	Married – partner present	62.7 (56.9-68.1)	611	69.8 (67.6-71.9)	4478
	Married – partner not present	34.7 (31.5-38.1)	1623	18.6 (15.4-22.2)	826
Pakistan (2017)	Not married	NA		NA	
	Married – partner present	44.4 (34.7-54.5)	190	50.9 (48.9-52.9)	5120
	Married – partner not present	34.7 (27.6-42.5)	403	28.2 (22.2-35.0)	378
**East Asia and the Pacific**					
Cambodia (2014)	Not married	40.3 (18.8-66.4)	16	38.6 (17.0-65.9)	22
	Married – partner present	54.3 (50.4-58.1)	1222	57.4 (55.5-59.2)	6638
	Married – partner not present	30.5 (22.8-39.4)	167	43.8 (34.2-53.9)	157
Indonesia (2017)	Not married	39.1 (22.4-58.7)	16	4.9 (1.1-19.9)	25
	Married – partner present	78.9 (75.7-81.7)	987	78.8 (78.0-79.5)	23 385
	Married – partner not present	38.4 (33.3-43.7)	610	64.5 (61.0-67.9)	1454
Myanmar (2015)	Not married	55.2 (11.2-92.4)	4	54.0 (21.1-83.7)	10
	Married – partner present	79.3 (74.6-83.3)	514	77.0 (75.2-78.7)	4449
	Married – partner not present	33.9 (26.3-42.4)	174	45.2 (37.5-53.0)	177
Papua New Guinea (2016)	Not married	17.3 (9.2-30.3)	75	19.6 (13.2-28.2)	141
	Married – partner present	53.7 (45.1-62.0)	356	49.1 (46.6-51.5)	5058
	Married – partner not present	47.0 (39.3-54.9)	454	41.2 (33.4-49.5)	422
Philippines (2017)	Not married	25.4 (15.3-39.1)	114	17.5 (10.1-28.5)	110
	Married – partner present	47.7 (41.8-53.7)	713	58.0 (56.4-59.7)	9206
	Married – partner not present	42.0 (30.7-54.3)	332	36.4 (30.5-42.8)	402
Timor Leste (2016)	Not married	NA	15	NA	20
	Married – partner present	43.9 (36.4-51.6)	212	48.5 (45.9-51.1)	3367
	Married – partner not present	23.2 (15.8-32.7)	142	21.5 (15.5-29.2)	225
**Latin America and the Caribbean**					
Colombia (2015)	Not married	83.8 (81.7-85.8)	2951	80.3 (77.3-82.9)	1740
	Married – partner present	84.2 (81.8-86.3)	2439	87.3 (86.3-88.3)	13 194
	Married – partner not present	84.0 (80.6-86.9)	870	75.5 (69.4-80.7)	334
Dominican Republic (2013)	Not married	73.5 (68.1-78.3)	612	66.1 (57.4-73.9)	265
	Married – partner present	83.3 (78.6-87.1)	844	83.9 (81.7-85.8)	2960
	Married – partner not present	68.6 (61.9-74.5)	303	71.9 (58.8-82.0)	82
Guatemala (2014)	Not married	75.6 (69.5-80.8)	313	57.0 (50.2-63.6)	308
	Married – partner present	64.8 (60.3-69.0)	621	67.9 (66.5-69.3)	9003
	Married – partner not present	55.2 (51.6-58.7)	1140	37.8 (32.6-43.3)	434
Haiti (2016)	Not married	36.9 (32.2-41.9)	651	34.1 (28.7-40.0)	444
	Married – partner present	44.6 (41.0-48.4)	872	48.5 (46.1-50.9)	2710
	Married – partner not present	34.6 (31.6-37.7)	1438	31.6 (26.8-36.8)	331
Honduras (2011)	Not married	75.7 (71.2-79.7)	589	70.6 (64.7-76.0)	314
	Married – partner present	76.0 (72.3-79.3)	844	78.3 (77.2-79.4)	8985
	Married – partner not present	55.8 (51.4-60.0)	672	51.7 (45.0-58.3)	281
Peru (2020)	Not married	NA		NA	
	Married – partner present	68.6 (65.1-72.0)	1471	66.7 (65.3-68.0)	11 877
	Married – partner not present	52.2 (45.4-59.0)	404	64.9 (56.4-72.5)	226

MHH – male-headed households, FHH – female-headed households, NA – not available, CI – confidence interval, mDFPS – demand for family planning satisfied by modern methods

*Source: Demographic and Health Survey 2010-2019.

Since we used mDFPS among all sexually active women when available and among married women only when information on never married women was not available, a comparison of mDFPS considering only married/in union women and considering all sexually active women, when available, is presented in the Table S3 in the **Online Supplementary Document**. The difference in mDFPS was lower than 5 percentage points in 39 of the 52 countries with available information.

### Reasons for non-use of modern contraceptives by sex of household head and marital status

When exploring the reasons for not using modern contraceptives, a clear pattern emerged. Currently married women who were not living in the same household than their husbands systematically reported infrequent sex more often than women not married or married and living with the partner. This pattern was even clearer among women living in FHH than in MHH. Among women in MHH, opposition by herself or others was also a frequently reported reason, especially among women living with their partners (**Table 3**).

**Table 3:** Reasons for non-use of modern contraceptives among women aged 15-49 years by region, sex of household head, and by marital status across 59 low- and middle-income countries*

		Reasons†							
		**Infrequent sex (%)**		**Other opposed (%)**		**Respondent opposed (%)**		**Other reasons (%)‡**	
**Region**	**Marital status**	**MHH**	**FHH**	**MHH**	**FHH**	**MHH**	**FHH**	**MHH**	**FHH**
West and Central Africa	Not married	19.1	20.1	9.1	6.0	11.3	12.2	38.0	42.9
	Married – partner present	12.0	18.2	17.2	15.0	14.2	16.2	39.2	34.0
	Married – partner not present	36.4	41.0	11.3	10.8	10.0	10.8	26.1	33.3
Eastern and Southern Africa	Not married	27.4	29.8	3.4	2.8	4.2	5.4	25.9	28.5
	Married – partner present	9.7	17.3	14.5	13.7	8.1	6.2	42.4	40.4
	Married – partner not present	42.3	47.7	8.1	8.7	4.5	4.5	27.6	25.9
Middle East and North Africa	Not married	NA	NA	NA	NA	NA	NA	NA	NA
	Married – partner present	16.5	55.0	13.3	7.4	7.5	2.7	57.9	30.1
	Married – partner not present	70.8	83.2	10.1	8.0	3.4	3.6	19.3	14.6
Europe and Central Asia	Not married	22.2	36.7	0.0	6.7	0.0	13.3	0.0	16.7
	Married – partner present	12.8	17.0	15.6	11.3	29.1	25.4	26.5	24.4
	Married – partner not present	46.9	54.8	6.9	15.6	22.3	11.6	15.4	21.2
South Asia	Not married	13.1	25.4	4.8	4.5	3.6	9.0	36.9	38.8
	Married – partner present	15.9	27.9	5.7	4.8	5.7	4.5	58.5	54.5
	Married – partner not present	57.2	64.8	3.2	3.6	4.3	3.6	27.7	25.0
East Asia and the Pacific	Not married	36.4	53.8	13.6	0.0	18.2	0.0	13.6	15.4
	Married – partner present	23.7	26.6	19.7	18.3	8.6	7.9	36.4	33.4
	Married – partner not present	53.1	54.9	15.5	13.0	4.7	3.8	16.3	15.9
Latin America and the Caribbean	Not married	40.3	35.7	5.6	4.4	13.3	10.2	21.2	33.6
	Married – partner present	19.8	16.6	11.3	8.5	11.4	12.9	45.7	45.6
	Married – partner not present	65.8	58.4	4.3	6.3	7.2	9.8	22.6	28.4

MHH – male-headed households, FHH – female-headed households

*Source: Demographic and Health Survey, 2010-2019.

†Respondents may have selected more than one reason.

‡Other reasons are lack of knowledge, lack of access, health concerns, method inconvenient to use, and fatalism.

The MENA region had the highest percentage of women reporting infrequent sexual activity as the reason for non-use of contraception. Among married women living in FHH without the husband present in the household, 83% reported infrequent sex as the reason for non-use, compared to 71% living in MHH. Among married women living with their partners in MENA countries, 55% of those living in HH reported infrequent sex, while 17% of those living in MHH did so. Infrequent sex was a less commonly reported reason in West and Central Africa (especially among married women with the husband living in the household, 18% in FHH and 12% in MHH). It was less frequently reported among unmarried women in West and Central Africa (20% in FHH and 19% in MHH) and in East Asia and the Pacific (25% and 13% in FHH and MHH, respectively) (**Table 3**).

Opposition by the woman or by others were the next two most reported reasons, both with a higher occurrence in MHH than in FHH in all countries studied. We found a high occurrence of non-use of modern contraceptives due to woman’s own opposition among women living with their partners in Europe and Central Asia (25% in FHH and 29% in MHH) while the highest occurrences of non-use due to opposition by others was among married women with the husband living in the household in South Asia (18% in FHH and 20% in MHH) (**Table 3****)**.

## DISCUSSION

We explored patterns and inequalities in the demand for family planning satisfied by modern methods among reproductive age women according to the sex of the household head and the marital status of the women and investigated some contextual characteristics of these households. We found higher mDFPS among women of reproductive age living in MHH compared to FHH in most countries. MHH also comprise of higher proportions of women of reproductive age who were married or in a union, living with their partners/husbands, and who were sexually active. These findings coincide with the lower proportions of women residing with their partners in MHH reporting infrequent sexual intercourse as the main reason for non-use of modern contraception compared to women in FHH. Among the non-users living with their partners in MHH, a high proportion were not using modern contraception due to other’s opposition regarding family planning.

West and Central Africa had different patterns of mDFPS according to the household head’s sex, with higher levels of mDFPS among women living in FHH. Besides lower mDFPS among women in MHH compared to FHH, the women in this region living in MHH had one of the highest proportions of reporting opposition by others as a reason for not using modern contraceptives. A previous study identified that most FHH in West and Central Africa are households where unmarried younger women live with children alone [28]. The higher levels of mDFPS among women living in FHH may largely be due to higher demand among unmarried sexually active women. These findings are aligned with a previous study that explored modern contraceptive use in married and unmarried young women and found that the region had a high proportion of unmarried sexually active adolescents, among which modern contraceptive use is much higher than among their married peers [20]. The lower mDFPS among women living in MHH is also corroborated by other studies showing extremely low levels of mDFPS among married women [17,20,29] and the opposition from partners identified as the main reason for the unmet need for family planning in the region [17]. Between countries, the larger gap was found in Guinea, where mDFPS among married women was extremely low. Less than 20% of the Guinean married women have their mDFPS [29] and, among only married adolescents, contraceptive use prevalence was lower than 8% [20].

We also identified that not married women from countries in Asia had lower levels of mDFPS compared to other regions and other categories. Despite the strong cultural Asian norms against premarital sex, there is evidence that it is becoming more common, particularly in the context of increasing age of marriage [30-32]. This evidence, combined with our findings, may indicate an inability of the Asian health services to provide family planning to unmarried women.

The literature exploring the association between sex of household head and family planning is scarce and mostly restricted to a single country or focused on adolescents and young adults. Two studies have explored the association between household headship and unmet need for contraception in Africa, one focused on married women and the other on adolescents. Their results are aligned with ours, with higher unmet need among women living in households headed by a woman than by a man [17,33]. This difference between women living in households headed by women and those headed by men may be partly explained by the high proportion of older female household heads, who may desire marital childbearing of their daughters or daughters-in-law and may be opposed to their use of contraceptives [33]. Furthermore, there may be a relationship between the contraceptive behavior of the household head and that of other family members. Two of the pathways by which parents may influence their children are parental modeling of sexual activity and parent-child sexual risk communication. The studies that attempted to explore the effect of household headship on sexual and reproductive health of children found higher teenage pregnancy among unmarried girls living in households headed by their mothers [34,35]. Higher levels of mother-daughter sexual risk communication were associated with fewer sexual intercourse episodes and higher self-efficacy in contraception use [36], but sexual behavior and the mother’s early pregnancy [37], as well as the lack of the protective paternal parenting [34], were associated with higher teenage pregnancy in FHH than in MHH.

Our study expands the analysis to 59 low- and middle-income countries from all world regions, considering a sample of all reproductive aged women. Using standardized definitions to compare mDFPS by household headship and marital status, also including the presence of the partner in the household and the time since the last sexual intercourse, we were able to better understand who the women in need for family planning services are and the main reasons for the differences we found regarding household headship and mDFPS.

Although mDFPS is a more sensitive indicator of family planning coverage, it still has some limitations in the definition of who are the women in need of contraceptives. The main limitation is related to women who are married or in a union, among which sexual activity is not considered. Previous studies have indicated that despite frequent sexual activity among married women is more common in African and Latin American countries, it is rare in several countries in Asia [38]. Our findings indicate that most of the married women who were not using contraceptives were not sexually active and, consequently, unable to get pregnant.

One limitation of our study is the way information on household headship is collected in DHS surveys. It is subjective and may led to misclassification as it depends on the respondent’s interpretation. In addition, we lack data on time since the woman became the household head and on other variables that would be relevant to explain the temporary absence of husbands or partners. Several factors are related to the formation of female household headship, such as death or disability of the husband, increased women’s life expectancy, economic difficulties that led the husband to work abroad, being abandoned by the husband, polygyny, local acceptability of single mothers, and female participation in economy [14,39]. Although women heads of households may experience several problems, as economic insecurity, task overload, and mental health problems [14], which may contribute to why FHH are viewed as vulnerable, yet there is evidence that FHH vulnerability is not dependent only on the sex of the household head but also on the presence of any adult male in the household [40]. FHH tend to be slightly poorer than MHH when there is no male in the household and they tend to be slightly wealthier when there is a male living in the household [40]. Regardless of household headship, wealth inequalities in mDFPS persist in several countries [6,9,29] and information on the context that led the household being headed by a woman could allow us to get to deeper explanations on the drivers of unsatisfied mDFPS among women living in FHH.

Additionally, to correctly understand our findings, it is also important to consider that the women with or without their demand for mDFPS are not necessarily the heads of the FHH. Our descriptive analysis indicated an average median age of the female household head older than 49 years and a high proportion of the female heads were not currently married. Therefore, many of the women who were the heads of the household were not included in our analysis because they were not interviewed in the women’s questionnaire. So, the women in FHH that are included in the analysis may be the head’s relatives, such as their daughters, sisters, in-laws, or non-related household members, besides the head.

## CONCLUSIONS

Our analysis indicated a relationship between sex of household head, marital status, sexual activity, and levels of mDFPS. In most countries, mDFPS was lower among women residing in FHH compared to women in MHH. This is possibly due to their reported less frequent sexual activity. A few countries presented a different pattern, indicating the variability in the trajectories that led the households be headed by women and the differences in societal norms that affect intra-household dynamics and expectations from women. While women from households headed by an older women may be unable to access family planning services due to sociocultural norms, in households that the head is a reproductive aged woman, the satisfaction of their demand for family planning may be influenced mainly by infrequent sexual activity, or emotional or economic insecurity.

Looking at mDFPS among unmarried and married women with and without the partner and the proportion of women who had sex in the last month, we bring attention to sexual inactivity among married women and the importance of rethinking the construction of the indicator of mDFPS to obtain a more realistic prediction of who does and does not have demand for family planning.

## Additional material


Online Supplementary Document
