# Supplementary material for: Women’s marital status and use of family planning services across male- and female-headed households in low- and middle-income countries
Source: J Glob Health. 2023 Mar 3;13:04015. doi: 10.7189/jogh.13.04015 (PMC9980282; doi:10.7189/jogh.13.04015)
Supplement: Online Supplementary Document [file jogh-13-04015-s001.pdf]

ONLINE SUPPLEMENTARY DOCUMENT

Title: Women's marital status and use of family planning services across male- and female-headed households in low- and middle-income countries

Authors: Franciele Hellwig, Ghada E. Saad, Andrea Wendt, Aluísio J. D. Barros

Table S1. Demand for family planning satisfied by modern methods (mDFPS) among women aged 15-49 years old in need of family planning, by sex of household head in 59 low- and middle-income countries. Source DHS, 2010-2019.

Country	level	mDFPS	
		% (95% CI)	N
**West & Central Africa**			
Benin (2017)	MHH	25.5 (24.0; 27.2)	4935
	FHH	24.5 (21.7; 27.4)	1115
Burkina Faso (2010)	MHH	37.5 (35.6; 39.4)	5394
	FHH	46.4 (40.6; 52.3)	439
Cameroon (2018)	MHH	37.0 (34.6; 39.5)	3366
	FHH	41.8 (38.4; 45.3)	1028
Chad (2014)	MHH	14.1 (12.5; 16.0)	3495
	FHH	17.7 (14.0; 22.2)	578
Congo Brazzaville (2011)	MHH	31.5 (29.1; 33.9)	4264
	FHH	39.1 (34.7; 43.7)	980
Congo Democratic Republic (2013)	MHH	16.0 (14.2; 17.8)	5607
	FHH	17.0 (14.3; 20.2)	1328
Cote d'Ivoire (2011)	MHH	27.3 (25.1; 29.7)	3160
	FHH	32.3 (27.8; 37.2)	686
Gabon (2012)	MHH	37.8 (35.2; 40.6)	2525
	FHH	43.1 (36.5; 49.9)	1277
Gambia (2019)	MHH	40.5 (37.8; 43.2)	2786
	FHH	34.5 (29.4; 40.1)	544
Ghana (2014)	MHH	39.5 (36.7; 42.3)	2621
	FHH	32.9 (29.1; 37.0)	1022
Guinea (2018)	MHH	22.9 (20.1; 26.0)	2515
	FHH	36.1 (30.0; 42.6)	456
Liberia (2019)	MHH	41.9 (38.3; 45.5)	2350
	FHH	49.7 (45.7; 53.6)	1291
Mali (2018)	MHH	40.9 (38.1; 43.7)	3321
	FHH	29.8 (24.6; 35.5)	451
Niger (2012)	MHH	29.4 (26.8; 32.1)	2671
	FHH	14.1 (10.3; 19.1)	303
Nigeria (2018)	MHH	30.9 (29.5; 32.3)	9980
	FHH	29.0 (26.2; 31.9)	1668
Senegal (2019)	MHH	53.0 (50.0; 56.1)	2030
	FHH	49.7 (44.2; 55.3)	740
Sierra Leone (2019)	MHH	47.4 (45.2; 49.5)	4508
	FHH	53.9 (51.0; 56.7)	1706

Togo (2013)	MHH	33.8 (31.5; 36.1)	3089
	FHH	35.3 (31.2; 39.6)	766
**Eastern & Southern Africa**			
Angola (2015)	MHH	25.8 (22.7; 29.0)	3835
	FHH	29.6 (25.4; 34.3)	1451
Burundi (2016)	MHH	38.5 (36.5; 40.5)	4977
	FHH	38.4 (34.8; 42.2)	832
Comoros (2012)	MHH	26.2 (23.1; 29.6)	1155
	FHH	26.2 (22.0; 31.0)	610
Ethiopia (2016)	MHH	61.1 (57.8; 64.2)	5278
	FHH	55.6 (50.0; 61.1)	819
Kenya (2014)	MHH	72.5 (70.7; 74.3)	5140
	FHH	64.1 (61.3; 66.8)	1973
Lesotho (2014)	MHH	76.7 (74.5; 78.8)	2569
	FHH	74.3 (69.7; 78.5)	691
Malawi (2015)	MHH	76.4 (75.3; 77.5)	10961
	FHH	57.9 (55.2; 60.5)	2321
Mozambique (2015)	MHH	50.6 (47.3; 54.0)	1905
	FHH	49.6 (45.5; 53.7)	852
Namibia (2013)	MHH	75.8 (73.4; 78.0)	1939
	FHH	80.8 (78.3; 83.0)	1684
Rwanda (2019)	MHH	73.2 (71.8; 74.5)	5137
	FHH	65.0 (61.3; 68.7)	982
South Africa (2016)	MHH	77.1 (74.5; 79.5)	2076
	FHH	73.7 (70.8; 76.4)	1708
Tanzania (2015)	MHH	53.0 (50.8; 55.2)	4761
	FHH	52.3 (47.9; 56.6)	933
Uganda (2016)	MHH	50.6 (48.8; 52.3)	6435
	FHH	49.5 (46.7; 52.4)	1876
Zambia (2018)	MHH	66.8 (64.9; 68.6)	5091
	FHH	55.0 (51.3; 58.5)	926
Zimbabwe (2015)	MHH	86.6 (85.0; 88.1)	3265
	FHH	80.2 (77.8; 82.3)	1789
**Middle East & North Africa**			
Egypt (2014)	MHH	80.6 (79.6; 81.5)	14204
	FHH	56.2 (49.9; 62.3)	349
Jordan (2017)	MHH	55.6 (53.8; 57.3)	8736
	FHH	36.9 (27.7; 47.1)	260
Yemen (2013)	MHH	40.9 (39.2; 42.6)	9395
	FHH	27.2 (20.2; 35.6)	290
**Europe & Central Asia**			
Albania (2017)	MHH	5.9 (5.0; 7.1)	4154
	FHH	7.0 (4.9; 9.9)	690
Armenia (2015)	MHH	39.8 (37.6; 42.1)	2141
	FHH	36.7 (31.7; 41.9)	582
Kyrgyzstan (2012)	MHH	62.9 (60.3; 65.4)	2482

	FHH	50.7 (45.0; 56.4)	465
Tajikistan (2017)	MHH	51.1 (48.7; 53.6)	3399
	FHH	46.2 (41.2; 51.3)	639
Turkey (2013)	MHH	60.2 (58.3; 62.0)	4983
	FHH	49.9 (43.3; 56.5)	295
**East Asia & the Pacific**			
Cambodia (2014)	MHH	57.0 (55.1; 58.8)	6816
	FHH	51.3 (47.5; 55.0)	1405
Indonesia (2017)	MHH	77.9 (77.1; 78.6)	24863
	FHH	63.1 (60.1; 66.1)	1613
Myanmar (2015)	MHH	75.8 (74.0; 77.4)	4635
	FHH	67.7 (63.2; 72.0)	692
Papua New Guinea (2016)	MHH	47.7 (45.4; 50.1)	5621
	FHH	47.2 (40.6; 53.9)	885
Philippines (2017)	MHH	56.7 (55.1; 58.3)	9718
	FHH	43.9 (38.8; 49.1)	1159
Timor Leste (2016)	MHH	46.6 (44.2; 48.9)	3612
	FHH	34.1 (28.4; 40.3)	369
**South Asia**			
Afghanistan (2015)	MHH	39.7 (37.7; 41.7)	13319
	FHH	14.2 (5.8; 30.8)	137
Bangladesh (2017)	MHH	74.8 (73.7; 75.8)	12326
	FHH	37.5 (34.6; 40.5)	1693
India (2015)	MHH	72.9 (72.6; 73.2)	309763
	FHH	60.5 (59.7; 61.4)	29934
Maldives (2016)	MHH	30.1 (26.6; 33.8)	1527
	FHH	27.0 (24.0; 30.3)	1236
Nepal (2016)	MHH	61.8 (59.7; 63.9)	5308
	FHH	42.4 (39.6; 45.2)	2234
Pakistan (2017)	MHH	49.4 (47.4; 51.3)	5498
	FHH	37.8 (32.4; 43.5)	593
**Latin America & the Caribbean**			
Colombia (2015)	MHH	86.3 (85.4; 87.2)	15268
	FHH	84.0 (82.6; 85.3)	6260
Dominican Republic (2013)	MHH	82.2 (80.1; 84.0)	3307
	FHH	77.3 (74.5; 80.0)	1759
Guatemala (2014)	MHH	66.3 (64.9; 67.6)	9745
	FHH	61.1 (58.5; 63.7)	2074
Haiti (2016)	MHH	45.1 (43.0; 47.1)	3484
	FHH	38.1 (35.9; 40.3)	2961
Honduras (2011)	MHH	77.3 (76.2; 78.3)	9579
	FHH	69.5 (67.0; 71.8)	2105
Peru (2020)	MHH	67.2 (65.9; 68.5)	12801
	FHH	67.2 (64.0; 70.1)	2507

Table S2. Descriptive characteristics of households according to sex of household head.

Country	household headship	Median age of the household head	Wome's median age	Currently married	Husband/partner present	Polygyny	Sex last month	Sex more than 6 months
**Middle East & North Africa**								
Egypt (2014)	MHH	45	33	97.9	NA	2.9	NA	NA
	FHH	58	36	42.3	0.0	3.2	0.0	0.0
Jordan (2017)	MHH	46	34	97.2	97.7	4.3	89.9	3.5
	FHH	58	**36**	47.1	58.6	8.9	29.0	57.5
Yemen (2013)	MHH	46	27	63.5	90.1	6.8	0.0	0.0
	FHH	52	28	32.5	43.2	7.0	0.0	0.0
**Eastern & Southern Africa**								
Angola (2015)	MHH	44	27	71.1	95.1	19.3	65.8	8.4
	FHH	43	28	32.9	61.7	36.9	38.6	24.2
Burundi (2016)	MHH	43	28	65.2	98.4	5.0	83.6	7.6
	FHH	47	28	29.3	17.2	12.7	22.5	43.6
Comoros (2012))	MHH	46	28	67.5	89.0	15.1	77.6	5.6
	FHH	44	28	53.4	62.0	21.0	55.1	19.5
Ethiopia (2016)	MHH	44	28	73.3	96.3	9.9	75.8	7.5
	FHH	45	28	40.0	43.4	27.1	32.7	41.1
Kenya (2014)	MHH	43	29	74.8	43.9	4.8	75.2	7.1
	FHH	46	30	39.0	9.9	12.0	34.1	25.9
Lesotho (2014)	MHH	47	29	71.1	57.3	1.9	53.9	8.1
	FHH	56	27	24.0	39.5	2.9	28.3	21.3
Malawi (2015)	MHH	41	28	78.6	95.8	10.8	75.4	6.7
	FHH	46	28	32.6	32.4	25.3	25.5	35.9
Mozambique (2015)	MHH	43	28	76.3	94.8	13.8	67.5	7.7

	FHH	44	29	42.1	47.5	25.9	38.9	21.8
Namibia (2013)	MHH	45	29	55.1	94.2	4.6	61.0	9.7
	FHH	48	29	21.2	52.5	9.1	35.5	19.8
Rwanda (2019)	MHH	43	29	63.2	98.4	6.1	82.2	9.2
	FHH	50	29	20.6	28.1	17.6	24.3	45.2
South Africa (2016)	MHH	47	30	57.6	96.8	1.9	69.0	6.7
	FHH	52	30	15.5	40.7	4.9	41.1	17.8
Tanzania (2015)	MHH	44	28	71.5	96.6	17.9	72.4	6.2
	FHH	50	30	28.7	60.3	37.9	40.8	20.6
Uganda (2016)	MHH	41	28	74.1	95.8	21.2	73.2	6.7
	FHH	46	29	36.2	32.1	42.3	33.6	27.2
Zambia (2018)	MHH	43	28	69.6	96.7	8.7	72.7	7.7
	FHH	47	29	19.7	40.0	31.4	29.2	29.5
Zimbabwe (2015)	MHH	43	28	72.4	94.3	7.1	80.7	5.7
	FHH	45	29	45.2	37.9	14.7	44.9	17.9
**West & Central Africa**								
Benin (2017)	MHH	43	28	77.1	94.9	35.8	60.5	8.6
	FHH	49	29	46.8	29.5	48.5	38.5	20.9
Burkina Faso (2010))	MHH	45	29	81.7	96.2	42.7	57.9	15.6
	FHH	46	29	47.2	14.5	32.3	23.4	38.3
Cameroon (2018)	MHH	44	28	66.0	94.8	20.8	68.5	7.3
	FHH	49	28	27.5	46.7	15.2	41.1	19.3
Chad (2014)	MHH	43	28	82.5	93.1	37.5	77.6	5.1
	FHH	45	29	49.2	30.0	45.4	29.4	36.0
Congo Brazzaville (2011)	MHH	43	29	75.0	96.9	13.5	69.0	5.5
	FHH	50	30	24.4	36.4	32.9	40.1	21.1
Congo DR (2013)	MHH	42	28	73.4	94.7	18.1	70.2	7.9
	FHH	46	29	42.6	28.0	46.2	36.7	27.5

Cote DIvoire (2011))	MHH	44	29	72.5	90.9	30.6	63.0	11.0
	FHH	49	28	32.1	25.7	34.4	40.4	24.2
Gabon (2012)	MHH	47	29	67.6	90.6	11.1	64.6	7.1
	FHH	51	29	36.8	28.1	19.9	47.7	14.2
Gambia (2019)	MHH	49	28	72.7	74.6	38.0	63.5	12.4
	FHH	48	28	49.1	25.2	34.0	33.4	34.7
Ghana (2014)	MHH	44	30	73.6	95.6	18.3	59.8	11.7
	FHH	47	30	29.8	14.3	22.7	28.2	31.3
Guinea (2018)	MHH	49	29	76.8	91.6	44.5	54.2	19.9
	FHH	51	28	48.0	34.8	37.1	33.1	35.3
Liberia (2019)	MHH	44	30	71.1	96.4	10.6	64.6	8.6
	FHH	45	29	34.9	62.2	17.5	47.6	15.4
Mali (2018)	MHH	47	28	82.3	94.8	32.9	76.7	6.6
	FHH	44	29	63.1	41.6	43.5	36.7	31.9
Niger (2012)	MHH	45	29	88.8	92.4	35.3	77.1	2.9
	FHH	46	28	61.1	13.3	28.0	12.7	32.4
Nigeria (2018)	MHH	45	29	76.4	96.3	30.6	74.8	7.0
	FHH	50	30	34.0	30.6	23.7	33.7	29.1
Senegal (2019)	MHH	54	28	71.6	73.5	30.8	63.8	11.4
	FHH	51	28	60.7	26.5	32.5	44.7	19.3
Sierra Leone (2019)	MHH	45	29	71.6	90.4	30.8	62.2	13.1
	FHH	47	28	42.0	40.9	34.5	44.8	18.1
Togo (2013)	MHH	44	29	76.5	94.7	32.4	59.9	12.0
	FHH	48	29	38.3	14.0	41.5	33.1	29.8
**Europe & Central Asia**								
Albania (2017)	MHH	58	32	71.9	96.4	0.0	81.7	7.1
	FHH	61	32	54.8	66.8	0.0	49.4	23.3
Armenia (2015)	MHH	56	31	70.4	97.4	0.0	90.2	3.6

	FHH	63	32	51.4	83.1	0.0	60.0	26.8
Kyrgyzstan (2012)	MHH	49	30	73.4	96.6	0.0	86.4	3.7
	FHH	56	30	43.5	82.2	0.0	50.0	33.4
Tajikistan (2017)	MHH	51	30	73.7	92.2	1.9	66.6	10.7
	FHH	54	30	57.5	76.1	6.2	43.8	33.1
Turkey (2013)	MHH	48	31	75.1	NA	NA	NA	NA
	FHH	58	31	33.6	NA	NA	NA	NA
**South Asia**								
Afghanistan (2015)	MHH	44	31	97.8	97.0	6.5	87.9	3.2
	FHH	47	31	59.2	62.0	3.3	36.9	48.3
Bangladesh (2017)	MHH	46	31	96.6	91.8	NA	82.5	6.0
	FHH	42	32	77.5	24.3	NA	31.3	39.3
India (2015)	MHH	48	30	74.4	95.3	1.7	69.0	5.3
	FHH	51	30	52.1	61.7	2.0	34.1	22.2
Maldives (2016)	MHH	53	31	74.8	86.2	1.0	73.1	6.4
	FHH	48	31	71.1	73.2	1.9	60.5	10.1
Nepal (2016)	MHH	48	29	78.3	82.2	3.9	73.8	8.9
	FHH	41	29	74.1	27.0	4.8	31.0	31.3
Pakistan (2017)	MHH	48	32	97.6	90.4	3.7	76.1	3.6
	FHH	47	33	85.1	29.1	2.8	45.0	15.2
**East Asia & the Pacific**								
Cambodia (2014)	MHH	46	30	71.9	97.3	2.0	81.6	4.5
	FHH	50	31	49.9	89.2	4.2	55.1	27.4
Indonesia (2017)	MHH	47	32	74.2	94.5	NA	79.4	4.5
	FHH	55	31	37.4	64.1	NA	37.5	43.3
Myanmar (2015)	MHH	49	31	67.6	95.0	5.2	75.4	6.9
	FHH	57	32	37.6	65.7	4.7	37.9	41.4
Papua New Guinea (2016)	MHH	44	29	69.8	92.2	15.2	61.5	15.7

	FHH	46	30	46.1	45.7	25.0	33.6	38.5
Philippines (2017)	MHH	47	30	67.5	94.7	100.0	71.1	8.5
	FHH	56	30	34.7	67.0	100.0	35.2	37.9
Timor Leste (2016)	MHH	49	29	63.8	94.2	3.7	68.3	10.6
	FHH	55	28	41.1	55.5	4.7	35.8	40.9
**Latin America & Caribbean**								
Colombia (2015)	MHH	49	30	69.9	97.3	NA	76.8	5.3
	FHH	51	31	30.8	72.0	NA	51.0	17.5
Dominican Republic (2013)	MHH	47	30	72.2	96.8	NA	81.7	3.8
	FHH	49	30	34.8	73.1	NA	49.6	19.0
Guatemala (2014)	MHH	46	29	64.9	95.0	NA	75.5	10.1
	FHH	48	29	38.4	31.9	NA	37.2	39.2
Haiti (2016)	MHH	49	28	59.5	88.1	8.2	66.8	8.0
	FHH	49	29	45.5	37.7	20.6	44.8	18.5
Honduras (2011)	MHH	45	29	69.1	96.4	NA	76.4	8.6
	FHH	50	29	29.1	54.6	NA	35.0	38.7
Peru (2020	MHH	47	31	71.8	97.6	NA	75.7	7.2
	FHH	48	31	33.0	74.7	NA	43.2	27.3

Table S3. Demand for family planning satisfied by modern methods among all sexually active women and among married/in union only.

Country	level	mDFPS (%)	
		married/in union	all sexually active women
**West & Central Africa**			
Benin (2017)	MHH	25.3	25.5
	FHH	22.4	24.5
Burkina Faso (2010)	MHH	36.4	37.5
	FHH	40.2	46.4
Cameroon (2018)	MHH	33.2	37.0
	FHH	34.4	41.8
Chad (2014)	MHH	13.8	14.1
	FHH	14.7	17.7
Congo Brazzaville (2011)	MHH	29.0	31.5
	FHH	30.5	39.1
Congo Democratic Republic (2013)	MHH	14.7	16.0
	FHH	15.2	17.0
Cote dIvoire (2011)	MHH	25.9	27.3
	FHH	23.3	32.3
Gabon (2012)	MHH	32.5	37.8
	FHH	34.0	43.1
Gambia (2019)	MHH	40.7	40.5
	FHH	31.6	34.5
Ghana (2014)	MHH	39.6	39.5
	FHH	33.0	32.9
Guinea (2018)	MHH	19.1	22.9
	FHH	24.3	36.1
Liberia (2019)	MHH	40.4	41.9
	FHH	41.0	49.7
Mali (2018)	MHH	40.9	40.9
	FHH	28.9	29.8
Niger (2012)	MHH	29.4	29.4
	FHH	12.2	14.1
Nigeria (2018)	MHH	31.0	30.9
	FHH	25.6	29.0
Senegal (2019)	MHH	53.1	53.0
	FHH	50.2	49.7
Sierra Leone (2019)	MHH	43.9	47.4
	FHH	48.7	53.9
Togo (2013)	MHH	33.1	33.8
	FHH	27.4	35.3
**Eastern & Southern Africa**			
Angola (2015)	MHH	23.9	25.8
	FHH	23.6	29.6

Burundi (2016)	MHH	38.4	38.5
	FHH	37.4	38.4
Comoros (2012)	MHH	25.9	26.2
	FHH	25.7	26.2
Ethiopia (2016)	MHH	61.1	61.1
	FHH	54.0	55.6
Kenya (2014)	MHH	72.6	72.5
	FHH	63.8	64.1
Lesotho (2014)	MHH	76.9	76.7
	FHH	70.9	74.3
Malawi (2015)	MHH	77.1	76.4
	FHH	59.0	57.9
Mozambique (2015)	MHH	49.0	50.6
	FHH	45.1	49.6
Namibia (2013)	MHH	75.2	75.8
	FHH	73.8	80.8
Rwanda (2019)	MHH	73.9	73.2
	FHH	66.7	65.0
South Africa (2016)	MHH	78.3	77.1
	FHH	76.0	73.7
Tanzania (2015)	MHH	52.8	53.0
	FHH	45.2	52.3
Uganda (2016)	MHH	50.5	50.6
	FHH	46.2	49.5
Zambia (2018)	MHH	67.8	66.8
	FHH	58.8	55.0
Zimbabwe (2015)	MHH	86.9	86.6
	FHH	80.7	80.2
**Europe & Central Asia**			
Albania (2017)	MHH	5.9	5.9
	FHH	6.6	7.0
Armenia (2015)	MHH	39.7	39.8
	FHH	36.1	36.7
Kyrgyzstan (2012)	MHH	62.9	62.9
	FHH	53.0	50.7
Tajikistan (2017)	MHH	51.2	51.1
	FHH	46.2	46.2
**South Asia**		0.0	0.0
India (2015)	MHH	72.9	72.9
	FHH	60.5	60.5
Maldives (2016)	MHH	31.0	30.1
	FHH	27.7	27.0
Nepal (2016)	MHH	61.8	61.8
	FHH	42.3	42.4
**East Asia & the Pacific**			
Cambodia (2014)	MHH	57.0	57.0

	FHH	51.4	51.3
Indonesia (2017)	MHH	77.9	77.9
	FHH	63.4	63.1
Myanmar (2015)	MHH	75.8	75.8
	FHH	67.8	67.7
Papua New Guinea (2016)	MHH	48.5	47.7
	FHH	49.9	47.2
Philippines (2017)	MHH	57.1	56.7
	FHH	45.9	43.9
Timor Leste (2016)	MHH	46.8	46.6
	FHH	35.6	34.1
**Latin America & Caribbean**			
Colombia (2015)	MHH	87.1	86.3
	FHH	84.2	84.0
Dominican Republic (2013)	MHH	83.6	82.2
	FHH	79.4	77.3
Guatemala (2014)	MHH	66.6	66.3
	FHH	58.6	61.1
Haiti (2016)	MHH	46.6	45.1
	FHH	38.4	38.1
Honduras (2011)	MHH	77.5	77.3
	FHH	67.0	69.5
Peru (2020)	MHH	66.6	67.2
	FHH	65.1	67.2
